# The Effects of Electron Beam Irradiation on the Morphological and Physicochemical Properties of Magnesium-Doped Hydroxyapatite/Chitosan Composite Coatings

**DOI:** 10.3390/polym14030582

**Published:** 2022-01-31

**Authors:** Bogdan Bita, Elena Stancu, Daniela Stroe, Mirabela Dumitrache, Steluta Carmen Ciobanu, Simona Liliana Iconaru, Daniela Predoi, Andreea Groza

**Affiliations:** 1 National Institute for Laser, Plasma and Radiation Physics, 409 Atomistilor Street, Magurele, 077125 Bucharest, Romania; bogdan.bita@inflpr.ro (B.B.); elena.stancu@inflpr.ro (E.S.); 2 Radiotherapy Department, Coltea Clinical Hospital, Ion C. Bratianu 1 Street, 030171 Bucharest, Romania; daniela.stroe@coltea.ro (D.S.); dumitrache.mira@gmail.com (M.D.); 3 Faculty of Physics, University of Bucharest, 077125 Magurele, Romania; 4 National Institute of Materials Physics, Atomistilor Street, No. 405A, 077125 Magurele, Romania; ciobanucs@gmail.com (S.C.C.); simonaiconaru@gmail.com (S.L.I.); dpredoi@gmail.com (D.P.)

**Keywords:** magnesium-doped hydroxyapatite/chitosan composite coatings, magnetron sputtering technique, electron beam irradiations

## Abstract

This work reports on the influence of 5 MeV electron beam radiations on the morphological features and chemical structure of magnesium-doped hydroxyapatite/chitosan composite coatings generated by the magnetron sputtering technique. The exposure to ionizing radiation in a linear electron accelerator dedicated to medical use has been performed in a controllable manner by delivering up to 50 Gy radiation dose in fractions of 2 Gy radiation dose per 40 s. After the irradiation with electron beams, the surface of layers became nano-size structured. The partial detachment of irradiated layers from the substrates has been revealed only after visualizing their cross sections by scanning electron microscopy. The energy dispersive X-ray spectral analysis of layer cross-sections indicated that the distribution of chemical elements in the samples depends on the radiation dose. The X-ray photoelectron spectroscopy, Fourier transform infrared spectroscopy and X-ray diffraction analysis have shown that the physicochemical processes induced by the ionizing radiation in the magnesium doped hydroxyapatite/chitosan composite coatings do not alter the apatite structure, and Mg remains bonded with the phosphate groups.

## 1. Introduction

Chitosan ((C_6_H_11_NO_4_)_n_) and hydroxyapatite (Ca_10_(PO_4_)_6_(OH)_2_) coatings, as well as their derived compounds, represent a class of material intensely studied in recent years for bone tissue engineering in biomedical applications [1,2,3,4]. In the research areas of orthopedics and cancer surgery [4,5], any type of calcium phosphate such as amorphous calcium phosphate, dicalcium phosphate, tricalcium phosphate, or octacalcium phosphate proved its utility for complex tissue repair and regeneration, reinforcement efficiency, bone engineering and therapeutic ions release. Significant importance is now given to producing implants or prostheses covered with bioactive layers based on hydroxyapatite (HAp) that can create strong interfacial bonds with the soft tissue [2]. The addition of chitosan to the hydroxyapatite layers improved the bioactivity and antimicrobial properties of the final compound, as well as its adhesion to substrates [2,3].

The surface chemical and biological activity of hydroxyapatite can be enhanced by doping the apatite structure with different ions such as Na^+^, K^+^, Mg^2+^, Sr^2+^, Ba^2+^, Al^3+^, Mn^2+^, Cu^2+^, Zn^2+^, Ag^+^, Ce^3+^, Eu^3+^, Sm^3+^), [6,7]. Mg^2+^ can perform cationic substitution with Ca, leading to Mg_3_(PO_4_)_2_ bonds formation [6,7]. Mg play an important role in the metabolism of the minerals into the muscle, nervous system and bones of the human body. Mg represents the main substitutes of calcium in the bone apatite structure being present in the human bones and teeth in a percentage higher than 50% [6,7,8]. The lack of Mg leads to the slowdown of bone development, osteoporosis, and reduces the activity of osteoblasts, osteoclasts and osteopenia cells [8,9].

The incorporation of Mg^2+^ ions into the hydroxyapatite structure changes the crystalline parameters of the synthesized compounds. These materials proved to be very helpful for bone mineralization by reducing bone fragility and enhancing the calcification process [8]. Nevertheless, the concentration of Mg^2+^ ions in Mg-doped hydroxyapatite powders needs to be carefully assessed in order to prevent toxicity to the human cells and any possible hemolytic activity [6,8]. Mg-doped hydroxyapatite powders have been produced by: sol-gel [6,7,8], co-precipitation [6], microwave-assisted wet chemical [8] or mechanochemical [9] synthesis methods. The synthesis of chitosan with Mg-doped hydroxyapatite can lead to the improvement of the final compound biocompatibility, bone regeneration, and antimicrobial properties [6,7].

Chitosan hydroxyapatite composite coatings are produced on various substrates by the following physical and chemical methods: electrophoretic deposition [10], spin coating technique [11], plasma spraying [12], electrochemical deposition [10] or radiofrequency magnetron sputtering discharges [13,14]. In our previous papers [13,14], we produced hydroxyapatite based layers by the radio frequency magnetron sputtering technique using sputtering targets composed of calcium phosphate [14], HAp doped with Ce^3+^ [15] and Sm^3+^ ions [16] or HAp synthesized with chitosan powders [13]. In the present study, we have shown that magnesium doped hydroxyapatite/chitosan composites layers can also be generated in radio frequency magnetron sputtering discharge.

Ionizing radiation such as X-rays or electron beams, produced in very well defined radiation dose conditions in medical linear accelerators, have beneficial effects in the treatment therapy of bone cancers such as pelvis cancer or pharyngeal cancer [17]. Direct ionization of the cells or the production of free radicals after the ionization or excitation of the water component of the cells represents the main microbiological effects of ionizing radiation on cancer cells, inducing their damage and death [18]. 

The radiation dose of few to tens of Gy’s are delivered to cancer-affected tissue in multiple exposure sessions established by a radiotherapy physician [17]. Usually, for pelvis cancer or pharyngeal cancer, the radiotherapy treatment starts with exposure, for a few seconds, to a 2 Gy electron beams absorbed dose and ends after the accumulation of 50 Gy of absorbed dose [17]. Moreover, radiotherapy treatments at low dosages (50 Gy accumulated dose delivered in 2 Gy dose fractions) for sterilization of any possible microscopic diseases developed after surgical treatments have been previously reported [19], and are currently used in hospitals. Besides, the ionizing radiation can significantly harm the healthy cells and tissue or possibly the interfacial layers between the orthopedic implants or prosthesis and tissue during the radiotherapy treatments. Several studies [20,21] showed the deterioration of bone formation, death, or decreased collagen of cell formation during and after radiotherapy treatments at an irradiation dose of tens of Gy [21].

The exposure of materials to gamma radiation or electron beams in the 10–50 kGy dose range is an accepted method worldwide for sterilization purposes [22,23]. However, compared to gamma radiation, due to their low penetration, the electron beams are used mainly for the sterilization of surfaces.

In our previous work [24], we have studied the modifications induced by a 2 Gy X-ray radiation dose on hydroxyapatite-polydimethylsiloxane composite layers. The depth profile analysis showed the depletion of Ca, P, C, O, Si, and H elements from the layers due to layer exfoliation. 

The present studies report on the results of the exposure of magnesium-doped hydroxyapatite/chitosan (MgHApCs) composite layers to electron beams of 5 MeV energies, produced in a linear accelerator dedicated to medical treatments of cancers by using ionizing radiation. We have investigated the effects of absorbed radiation doses on physicochemical and morphological properties of the composite layers. The irradiations of MgHApCs layers with electron beams have been performed following a complete radiotherapy scheme (in terms of radiation dose per time delivering fractions) used to treat pelvis cancer in accordance with the medical and clinical protocols [17].

The MgHApCs layers have been generated in radio frequency magnetron sputtering discharge. The physicochemical modifications generated by the electron beams (produced at 2 Gy respectively 50 Gy radiation dosages) in the layer bulks and at their surfaces were investigated by Fourier transform infrared spectroscopy (FTIR), X-ray photoelectron spectroscopy (XPS) and X-ray diffraction (XRD). The morphological surface features of unirradiated and irradiated MgHApCs layers were analyzed by scanning electron microscopy (SEM). By energy-dispersive X-ray spectroscopy (EDS), the elemental composition and depth profiles of unirradiated and irradiated layers have been performed. The adhesion strength of the layers to substrates was tested by using standard adherence tapes.

## 2. Materials and Methods

### 2.1. Materials

In order to obtain magnesium doped hydroxyapatite (Ca_10−x_Mg_x_(PO_4_)_6_(OH)_2_; x_Mg_ = 0.1; (Ca + Mg)/*p* = 1.67) in chitosan matrix, calcium nitrate tetrahydrate (Ca(NO_3_)_2_∙4H_2_O, Sigma Aldrich, St. Louis, MO, USA, ≥99.0%), magnesium nitrate hexahydrate (Mg(NO_3_)_2_·6H_2_O, Alpha Aesar, Kandel, Germany, 99.97% purity), ammonium hydrogen phosphate ((NH_4_)_2_HPO_4_, Sigma Aldrich, St. Louis, MO, USA, ≥99.0%) ammonium hydroxide (NH_4_OH, Sigma Aldrich, St. Louis, MO, USA, 25% NH_3_ in H_2_O (T)), chitosan (Sigma Aldrich, St. Louis, MO, USA) and double distilled water were used as precursors without further purification.

### 2.2. Synthesis of Magnesium-Doped Hydroxyapatite/Chitosan Composite Powders

The magnesium-doped hydroxyapatite/chitosan (MgHApCs) composite powders were obtained using an adapted co-precipitation method [1,25]. The atomic ratio used for Mg/[Mg + Ca] was equal to 10% and for [Ca + Mg]/P was equal to 1.67. The synthesis process took place in environmental conditions. Thus, by dissolving the adequate quantities of (NH_4_)_2_HPO_4_ in 300 mL double distilled water, solution 1 was obtained. Moreover, by dissolving the proper quantities of Ca(NO_3_)_2_·4H_2_O, Mg(NO_3_)_2_·6H_2_O and chitosan (ratio 3:2) in 300 mL double distilled water, solution 2 was obtained. Then, solution 2 was added drop by drop to solution 1 under vigorous stirring (400 rpm). In order to maintain the pH value at 10, NH_4_OH solution was used. The obtained mixture was washed and filtered five times consecutively. The final precipitate was dried at 150 °C (in an oven) in the air to obtain the magnesium-doped hydroxyapatite/chitosan composite powders. The MgHApCs powders were used to obtain the targets (two inches in diameter) by mechanical pressing. Finally, the MgHApCs targets were thermally treated in a furnace at 150 °C (5 °C/min) in air.

### 2.3. Synthesis of Magnesium-Doped Hydroxyapatite/Chitosan Composites Layers

Magnesium doped hydroxyapatite/chitosan layers have been generated by radio-frequency (RF) magnetron sputtering technique in Argon working gas on Si substrates in the following experimental conditions: 70 W RF power; 5 × 10^−3^ mbarr Ar gas pressures (base pressure ~10^−5^ mbarr); 2 sl_n_/min Ar gas flow; 4 cm magnetron source–substrate holder distance and 6 h deposition time. The layer thickness was calculated to be ~300 nm from the deposition rate measurements performed by using a quartz microbalance (acquired from INFICON Holding AG Company, Bad Ragaz, Switzerland) positioned on the holder substrate in the central position relative to the magnetron source. Additional details about the deposition technique, experimental set-up and deposition rate measurements can be found in [13,14].

### 2.4. Electron Beam Irradiation of Magnesium Doped Hydroxyapatite/Chitosan Composite Layers

The MgHApCs layers were irradiated using a Siemens MEVATRON Primus clinical linear accelerator. The samples were placed at 100 cm from the focus and were irradiated with 5 MeV electron beam and dose rate of 3 Gy/min with an error percentage of 5%. To provide homogenous irradiation of all samples, a large beam field of 20 cm × 20 cm has been used. The absorbed dose of 2 Gy per fraction (40 s) and 50 Gy total absorbed dose (25 fractions) was calculated following the medical radiation therapy protocols [17]. These electron beam dosimetry parameters were calculated and measured in the irradiation plane of samples in order to simulate as much as possible a clinical case: a radiotherapy complete treatment scheme for a patient with a prosthesis covered with an MgHApCs layer.

### 2.5. Characterization Methods

The surfaces and cross-sections of the irradiated and unirradiated MgHApCs layers deposited on Si substrates were analyzed by using a ThermoFisher Apreo S scanning electron microscope in both high- and low-vacuum modes. The EDAX Inc. SiLi detector has proved its powerful capacities in in-depth profile elemental analysis of unirradiated and irradiated samples. The dispersive energy X-ray (EDS) spectra of unirradiated and irradiated MgHApCs layers have been acquired at different high voltages applied on the field emission gun (FEG), between 2–10 kV.

The adherence of the unirradiated and irradiated MgHApCs composite coatings to silicon substrates was investigated in accordance with the ASTM standard procedure [26] for adhesion tape tests (D3330 test method for peel adhesion of pressure-sensitive tape). A 3M Performance Flatback Tape 2525 tape with a peel adhesion of 7.5 N/cm and tensile strength of 85.8 N/cm was used. In order to analyze only the adhesion strength of the layers with minimal influence from the roughness of the substrate surface, we used silicon substrates with mirror-like surfaces.

XPS analyses of the surfaces of unirradiated and irradiated MgHApCs coatings were performed by using a K-alpha Thermo Scientific (ESCALAB™ XI+, East Grinstead, UK) spectrometer equipped with a 180° double-focusing hemispherical analyzer. The survey spectra of the layers were acquired with a pass energy of 50 eV. The position of C 1s peak was at 284.8 eV. To identify physicochemical modifications induced on the layer surfaces by the electron beams, the high-resolution spectra of XPS lines were recorded with 20 eV pass energy and 0.1 eV energy step size. Advanced Advantage data software has been used to acquire and process XPS spectra. 

In the 4000–400 cm^−1^ IR spectral range, we recorded the attenuated total reflection IR spectra of unirradiated and irradiated MgHApCs layers utilizing a Perkin Elmer SP-100 spectrometer (Waltham, MA, USA). By the curve-fitting procedure described in [27], we performed spectral deconvolutions in the 1200–800 cm^−1^ range specific to P-O bonds employing MagicPlot software.

The crystalline structure of the MgHApCs thin films was examined by X-ray diffraction (XRD). The MgHApCs thin film was measured in grazing incidence (GIXRD) geometry, with a Rigaku SmartLab 3 kW equipment, using CuKα radiation (λ = 1.5418 Å) and an incidence angle of 0.5° [28]. The patterns were acquired in the 2θ range 15–70°, with a step size of 0.02°, and a dwell time of 8.5 s.

## 3. Results and Discussions

### 3.1. SEM and EDS Analysis of Magnesium Doped Hydroxyapatite/Chitosan Composite Layers

The exposure of MgHApCs coatings to electron beams of different radiation doses induces morphological and structural modifications at the layer surfaces and their volumes. 

**Figure 1:**
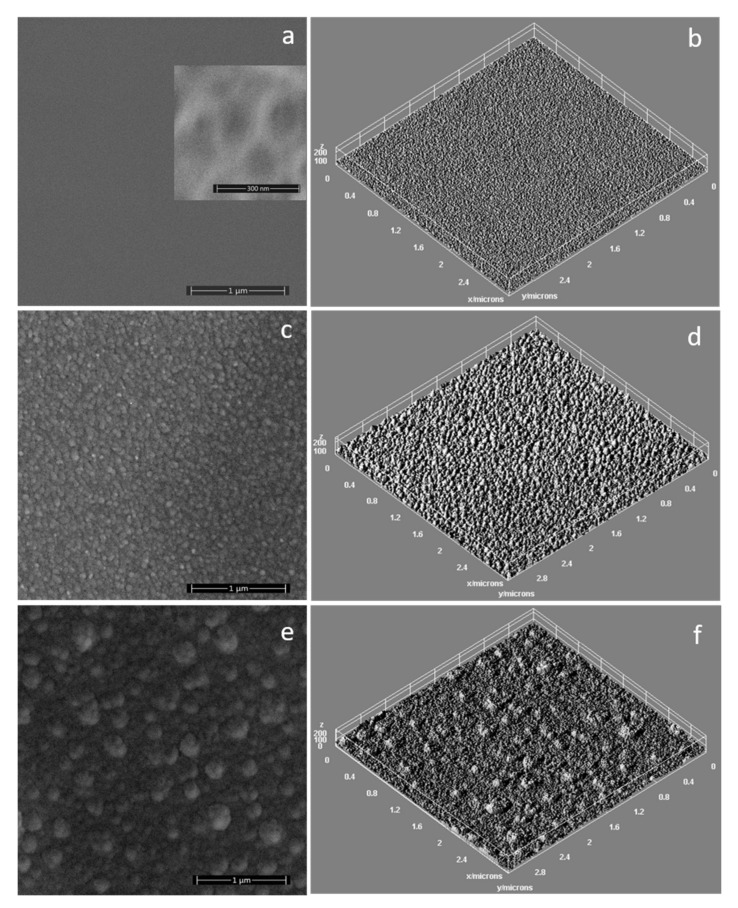
SEM images of: (**a**) unirradiated; (**c**) 2 Gy irradiated; (**e**) 50 Gy irradiated; MgHApCs layers. 3D surface plot of SEM images of: (**b**) unirradiated; (**d**) 2 Gy irradiated; (**f**) 50 Gy irradiated; MgHApCs layers.

The surface of the unirradiated layer is smooth, and its pattern (see caption inserted in Figure 1a), is due to the polymeric molecules sputtered from the target (during the deposition process in the rf magnetron discharge) that condensate when they reach the substrate. In comparison with our previous results regarding the deposition of HApCs layers in similar experimental conditions [13], the adding of Mg into the composition of the sputtering target and the increasing of applied rf power lead to the smoothing of the layer surface [13].

In Figure 1c,e are presented the SEM images that illustrate the topography of the surfaces of MgHApCs coatings after irradiation with electron beams of 2 and 50 Gy radiation doses. We attributed the surfaces’ nano-sized structuring to the shrinkage of the polymer content from the layers. The size of the structures increases with the radiation dose (see Figure 1c,e) due to the coagulation of small structures into bigger ones during the multiple electron beam exposure sessions. Better visualization of the complex surface textures of the irradiated layers was assessed by the 3D surface plots presented in Figure 1b,d,f and obtained by using Image J software (ImageJ 1.51j8, National Institutes of Health, Bethesda, MD, USA).

**Figure 2:**
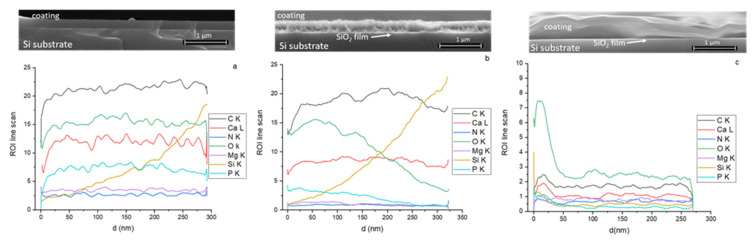
SEM depth profiles and cross-section images of: (**a**) unirradiated; (**b**) 2 Gy irradiated; (**c**) 50 Gy irradiated; MgHApCs layers. EDS depth profiles of (**a**) unirradiated; (**b**) 2 Gy irradiated; (**c**) 50 Gy irradiated; MgHApCs layers.

The SEM images of the MgHApCs layer cross-sections (see captions from Figure 2a–c) show the effects of electron beam irradiations at the layer/substrate interface. As the value of the irradiation dose increases, the layers are gradually detached from the substrate. Moreover, at the interface between Si substrate and MgHApCs coating, a thin silicon oxide layer is observed to be formed [29].

The EDS line scan of the coating cross-sections [30] allowed the evaluation of the distribution of the C, N, O, Ca, P, Mg elements into the layer volume. In Figure 2a–c are shown the depth profile of each element contained in the unirradiated and, respectively, the irradiated layers. The signals have been collected from the layer surface up to the Si substrate interface for 5 kV voltage applied on the SEM FEG. The smooth behavior of Ca, P, C, O, Mg and N signals indicate a homogenous distribution of Ca, P, C, O, Mg and N elements in the volume of the unirradiated MgHApCs layer (see Figure 2a).

The elemental depth profiles of Ca, P, C, O, Mg and N elements are modified as the electron beam radiation dose increases from 2 to 50 Gy. In Figure 2b can be observed that the decreasing of the intensity of the Ca, P, O, Mg and N, depth profiles at Si substrate interface are accompanied by the increase of the Si signal intensity in the layered bulk, indicating the begging of the layer detachment. After the absorption of 50 Gy dose, the MgHapCs layer is squeezed and almost completely removed from the substrate surface (see Figure 2c). The multiple scanning of the layer cross-sections shows that portions of the removed layer alternate with the portions where the layers are still bonded to the substrates.

The element depth profiles evolution in the irradiated samples also indicates the incorporation of Si into the layers (see Figure 2b,c). Therefore, we assume that during the electron beam irradiations, the diffusion of Si into the layer volume takes place. Because the shape of the Si depth profile is similar to that of the Ca, P, C, O, Mg and N elements in the 50 Gy irradiated MgHApCs sample (see Figure 2c), we suppose that the elements depleted/redistributed into the layers could be linked with the layer shrinkage and its partial detachment from the substrate.

**Figure 3:**
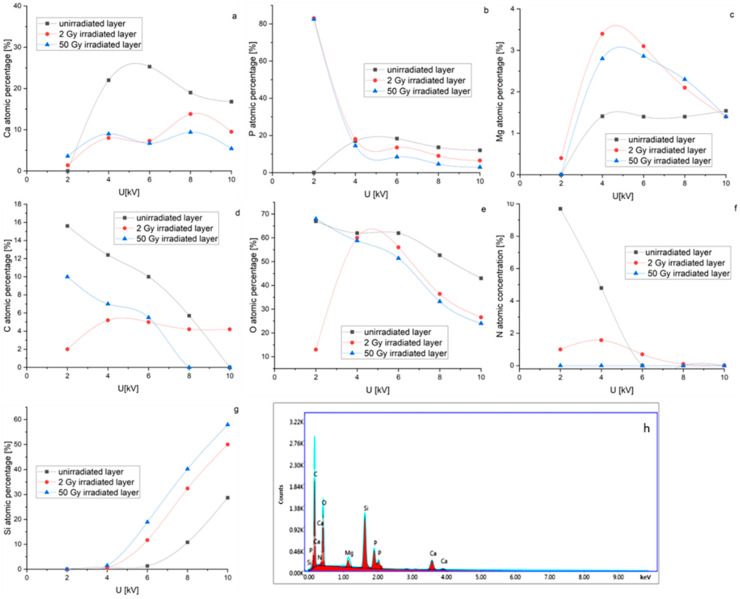
Evolution of atomic percentage of the following elements: (**a**) Ca; (**b**) P; (**c**)Mg; (**d**) C; (**e**) O; (**f**) N; (**g**) Si in unirradiated and irradiated MgHApCs coatings deposited on Si substrates. 3(**h**) EDS spectrum of unirradiated MgHApCs layer.

The EDS quantitative analysis of unirradiated and irradiated MgHApCs layers accomplished at different applied voltages on SEM FEG (see Figure 3a–g) allowed us to follow the evolution of the atomic percentages of the Ca, P, C, O, Mg and N chemical elements as a function of radiation dose. As the applied voltage on the SEM FEG increases, the depth of electron penetration into the sample increases [31]; therefore, the number of atoms from the sample subjected to electron beams emit X-rays and the atomic percentages of the elements increases.

The Ca, P, C, O, Mg and N atomic percentage curves are studied compared to the silicon atomic percentage curve (see Figure 3a–g). The evolution of Ca, P and Mg atomic percentage curve shapes with the applied voltage is similar in the unirradiated samples (see Figure 3a–c), which could denote that the atoms are bonded in the same chemical structure. In the irradiated samples, the shapes of Ca, P and Mg atomic percentage curves suggest structural modifications. Moreover, decreasing of O, C and N atomic percentages at low voltages (see Figure 3d–f) infer their depletion due to layer shrinkage.

In Figure 3g it can be observed that Si atomic percentage is firstly detected at 6 kV in the unirradiated sample. With the increase of the irradiation dose, the Si atomic percentage is recorded at lower voltages, implying that the layer thickness decreases or the Si diffuses into the MgHApCs layer. These suppositions are sustained by the SEM images of the cross-sections of unirradiated and irradiated layers (see Figure 2), which indicate the shrinkage of the polymer and layer detachment at the 50 Gy irradiation dose. 

The EDS spectrum of the MgHApCs unirradiated layer is presented in Figure 3h.

### 3.2. SEM Analysis of Magnesium Doped Hydroxyapatite/Chitosan Composite Layers Adhesion by Tape Test

**Figure 4:**
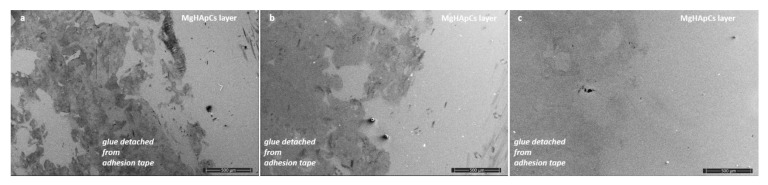
SEM images of adhesion tape tests of: (**a**) unirradiated MgHApCs; (**b**) 2 Gy irradiated MgHApCs; (**c**) 50 Gy irradiated MgHApCs; layers.

The adhesion strength of MgHapCs layers to the mirror-like surfaces of the Si substrates was investigated by SEM. In Figure 4a–c it can be observed that both the unirradiated and irradiated layers after the pealing of the 3M Performance Flatback Tape 2525 adhesive tape are not exfoliated. On the other hand, the glue from the adhesive tape remained stuck to the surface of the layers as an indication that these layers could be used as interfacial layers.

### 3.3. XPS Analysis of Magnesium Doped Hydroxyapatite/Chitosan Composite Layers

The surface chemistry of the MgHApCs coatings before and after the electron beam irradiations has been studied by X-ray photoelectron spectroscopy (XPS). We concentrate our analysis on the influence of the radiation dose values on the Mg linkage with the phosphate group and the chemical bonding in chitosan.

**Figure 5:**
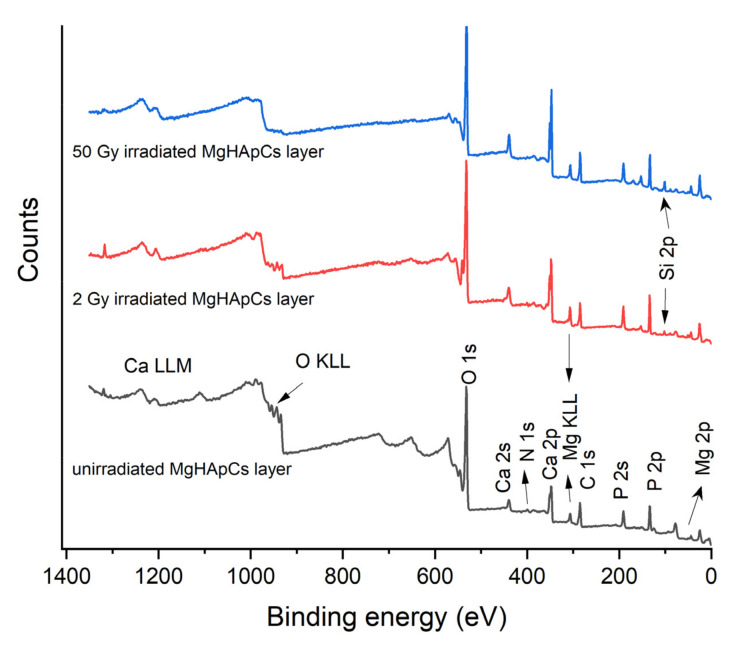
XPS Survey spectra of unirradiated and irradiated MgHApCs layers.

The Ca, P, O, N, C, Mg elements have been detected in the XPS full spectrum of unirradiated MgHApCs layer shown in Figure 5. In the XPS spectra of 2 Gy and 50 Gy irradiated MgHApCs coatings (see Figure 5) were identified Si 2p XPS peaks. We assume that the silicon presence on the surface of irradiated layers can be attributed to the silicon oxide formation at the layer/substrate interface and consecutively to the Si diffusion into the layer volume.

**Figure 6:**
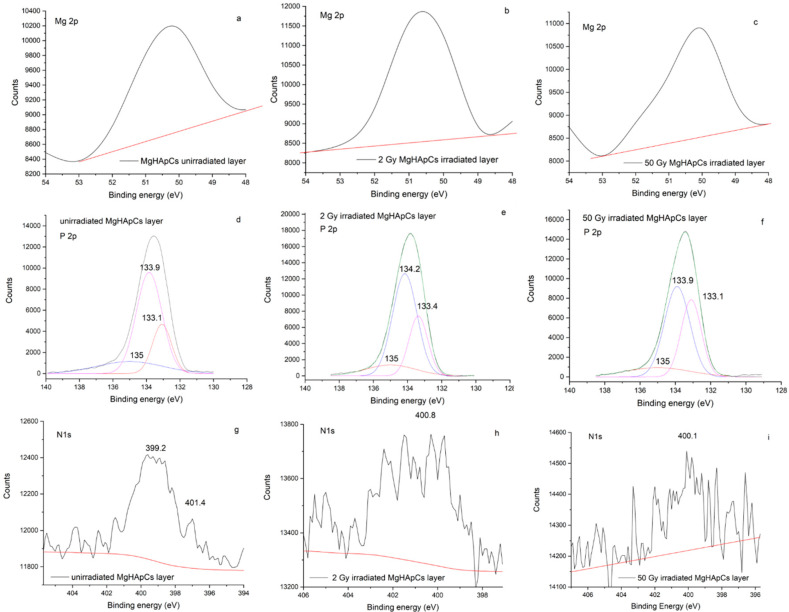
High-resolution XPS lines of: (**a**–**c**) Mg 2p in unirradiated and irradiated MgHApCs samples; (**d**–**f**) P 2p in unirradiated and irradiated MgHApCs samples; (**g**–**i**) N 1s in unirradiated and irradiated MgHApCs samples.

The Mg 2p XPS peaks for all the analyzed samples are exhibited in Figure 6a–c. Their presence at 50 eV (for unirradiated MgHApCs sample), 50.5 eV (for 2 Gy irradiated MgHApCs sample), and 50 eV (for 50 Gy irradiated MgHApCs sample) indicate that the embedding of Mg into the HAp structure and formation of Mg_3_(PO_4_)_2_ chemical bonds [32] is not altered by the exposure to electron beams.

The high-resolution P 2p XPS peaks (see Figure 6d–f) of unirradiated and irradiated MgHApCs layers are deconvoluted in three components. The 133.1 eV P 2p_3/2_ XPS and 133.9 eV P 2p_1/2_ XPS peaks indicate the P-O bonds in (PO_4_)^3−^ groups. The 135 eV deconvoluted peak ascertain the Mg_3_(PO_4_)_2_ bonds formation [33]. P 2p_3/2_ and P 2p_1/2_ XPS peaks are slightly changed in the 2 Gy and 50 Gy irradiated samples. Nevertheless, the 0.8 eV difference between P 2p_3/2_ and P 2p_1/2_ XPS peaks [13,34] is maintained as an indication that the exposure to ionizing radiation does not destroy the apatite structure of the samples. 

The high-resolution N 1s XPS spectrum of the unirradiated MgHApCs layer shows two peaks at 399.2 eV and 401.2 eV (see Figure 6g). Previously, these peaks were also identified in the XPS spectrum of chitosan [35]. The first peak was attributed to non-protonated amine or amide groups, while the second peak was assigned to protonated amine [35]. In the XPS spectra of 2 Gy and 50 Gy irradiated layers (see Figure 6h,i), the position of N 1s XPS peaks is slightly moved to higher energies, suggesting structural modifications in chitosan, namely the protonation of amine/amide groups [35]. The intensity of the N 1s XPS peak decreases as the irradiation dose increases, possibly due to the depletion or redistribution of nitrogen atoms into the layers.

### 3.4. Fourier Transform Infrared Spectroscopy Analyses of Magnesium-Doped Hydroxyapatite/Chitosan Composite Coatings

The chemical bonds of hydroxyapatite that absorb the infrared radiation and manifest their vibrational modes are the [PO_4_]^3−^groups. The vibrations of the P-O bonds in the [PO_4_]^3−^groups depend on the physical state of hydroxyapatite (crystalline or amorphous) and on its chemical state (doped/undoped/composites). Usually, P-O bonds have the following vibrational modes in [PO_4_]^3−^groups: 1200–900 cm^−1^ (ν_3_), 960 cm^−1^ (ν_1_), 630–500 cm^−1^ (ν_4_), 470 cm^−1^ (ν_2_) [13,14,36]. 

The position of the (ν_3_), (ν_1_) and (ν_4_) frequencies in the IR spectra are the ones most influenced by the hydroxyapatite doped/undoped or composite character. In our previous work [13], by analyzing the molecular features of chitosan hydroxyapatite composite coatings produced in rf magnetron sputtering discharge, we identified the ν_1_ vibrational mode of P-O bonds in [PO_4_]^3−^ groups at 940 cm^−1^.

**Figure 7:**
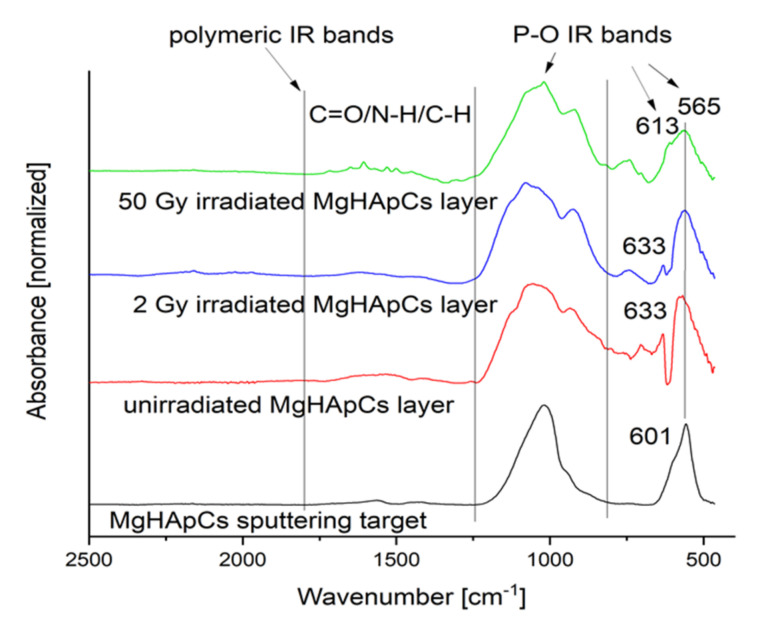
FTIR absorbance spectra of sputtering target, unirradiated and irradiated MgHApCs layers.

Figure 7 presents the IR absorption spectra of unirradiated and irradiated MgHApCs coatings and the sputtering target. At first look, it seems that the electron beams do not produce significant modifications on the molecular structure of hydroxyapatite and chitosan, as their characteristic vibrational bands [37,38] are observed, and new vibrational IR bands do not appear. There are no differences between the IR bands specific to chitosan from Figure 7 and those previously reported in [13].

**Figure 8:**
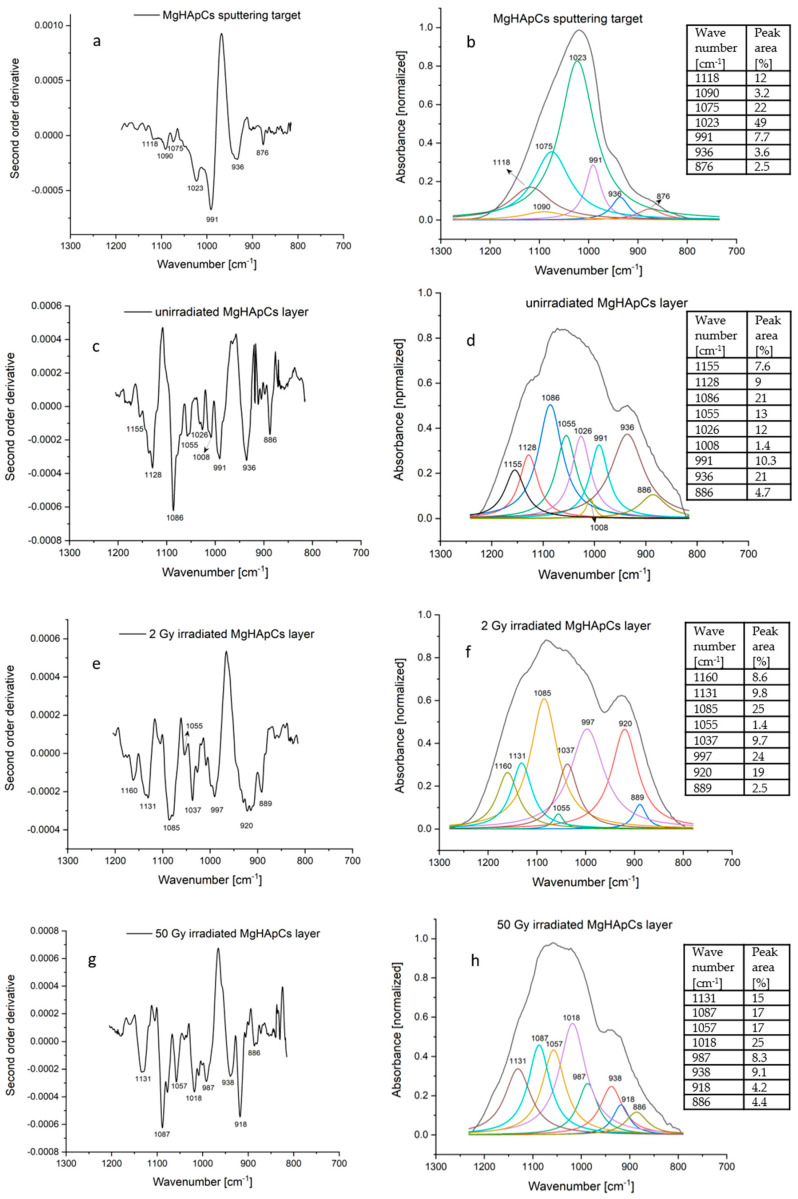
Second order derivative of: (**a**) sputtering target; (**c**) unirradiated; (**e**) 2 Gy irradiated; (**g**) 50 Gy irradiated; MgHApCs layer. Deconvoluted IR bands of: (**b**) sputtering target; (**d**) unirradiated; (**f**) 2 Gy irradiated; (**h**) 50 Gy irradiated; MgHApCs layer.

In this context, the curve fitting analysis in the 1200–800 cm^−1^ spectral range could reveal the modifications of the molecular structure of MgHApCs coatings and, consecutively, the frequencies of P-O bond vibrations in the IR spectra of the sputtering target, and the unirradiated and irradiated MgHApCs layers (see Figure 8 and Table 1).

The coatings’ complex composite structure leads to the superposition of IR absorption bands characteristic both to magnesium doped hydroxyapatite and chitosan. Figure 8 and Table 1 report the IR spectral fitting analysis results and the correspondence between the wavenumbers of deconvoluted absorption bands and vibrational bonds. We also calculated the peak area percentages of deconvoluted IR bands.

For accuracy, Figure 8a,c,e,g present the second-order derivatives of the IR spectra bands of MgHapCs target, unirradiated and irradiated MgHApCs coatings. By analyzing the deconvoluted IR bands from Figure 8b,d,f,h, the intensity distribution of the deconvoluted IR bands is similar to that of the second-order derivatives. 

The IR deconvoluted bands from 1155 cm^−1^ (in the IR spectra of unirradiated MgHApCs layer) shifts to 1160 cm^−1^ in the 2 Gy irradiated MgHApCs layer IR spectrum and could be assigned either to P-O or Si-O bond vibrations [38]. The molecular band from ~920 cm^−1^ identified in the deconvoluted spectra of irradiated samples (see Figure 8f,h and Table 1) can be attributed to Si-OH bonds formation. These results are sustained by the EDS depth profile from Figure 2 and XPS survey spectra from Figure 5, which signals the presence of Si at the surface and in the volume of irradiated layers. 

The IR band from 1118 cm^−1^ (see Figure 8b) assigned to P-O vibrations in [PO_4_]^3−^ groups of the sputtering target is shifted to 1128 cm^−1^ in the IR spectrum of the unirradiated MgHApCs layer, and up to 1131 cm^−1^ in the IR spectra of the 2 Gy and 50 Gy irradiated MgHApCs layers. Similar shifts of P-O bond vibrational frequencies were observed in the case of HAp heated at hundreds of degrees [36].

Previously, in [37], a method was reported to evaluate the apatite/non-apatite structure of a calcium phosphate compound as a function of peak area ratio between P-O IR absorption bands. The overlapping of hydroxyapatite molecular bands with those of chitosan and the finding of new bands assigned to Si-O bond vibrations make the accuracy of such calculations from the deconvoluted IR spectra very difficult. Therefore, the peak area percentages of each deconvoluted IR band can be used only for a qualitative estimation of the processes encountered in the hydroxyapatite and chitosan molecular structures. Also, the broadening/narrowing of the ν_1_–ν_3_ PO_4_ (1200–900 cm^−1^) IR bands with the increasing radiation dose from 2 to 50 Gy can be observed in Figure 8f–h. The narrowing of the ν_1_–ν_3_ PO_4_ (1200–900 cm^−1^) IR bands at 50 Gy irradiation dose suggest possible crystallization.

Thus, it can be concluded that electron beams induce redistribution and rearrangements of phosphorus and oxygen in the apatite structure, without major changes in the molecular structure of chitosan.

### 3.5. X-ray Diffraction Analyses of Magnesium-Doped Hydroxyapatite/Chitosan Composite Coatings

**Figure 9:**
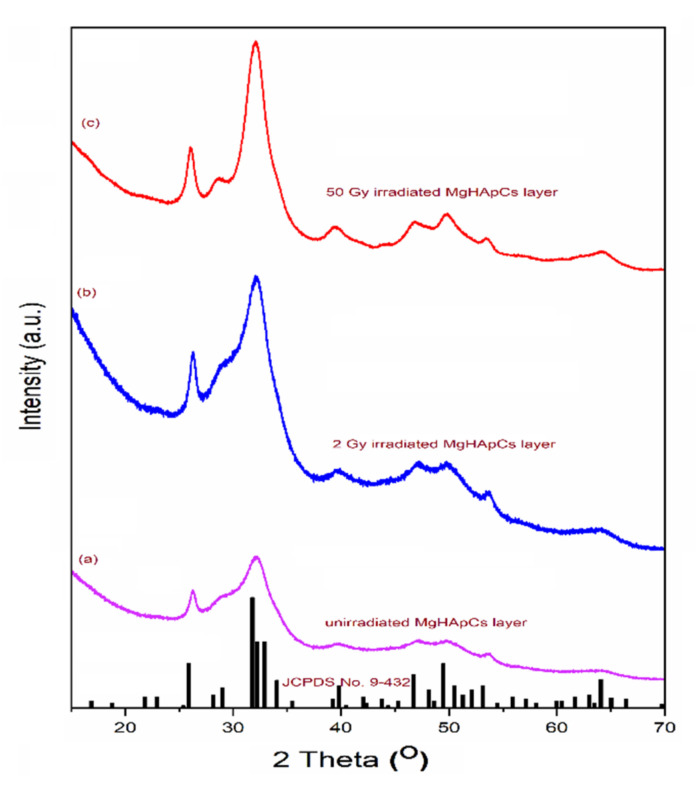
XRD patterns of the: (**a**) unirradiated; (**b**) 2 Gy irradiated; (**c**) 50 Gy irradiated; MgHApCs layers.

Figure 9 presents the XRD patterns of the unirradiated (a), 2 Gy irradiated (b) and 50 Gy irradiated (c) MgHApCs layers. The diffraction peaks were in good agreement with the standard card of the HAp (ICDD-PDF #9-0432). The influence of the electron beam irradiation of the MgHApCs layers was highlighted. Thus, the peak broadening of the MgHApCs layers decreased with the increase of the irradiation dose, demonstrating the fact that thin films irradiated at 50 Gy have a better-structured surface (see Figure 9c).

## 4. Conclusions

Magnesium-doped hydroxyapatite/chitosan composite layers have been generated in an rf magnetron sputtering discharge on Si substrates. The irradiation of the layers with electron beams of 2 Gy and 50 Gy radiation doses conduce to morphological and physicochemical modifications. The layer surfaces became nano-sized structured as a function of the value of the ionizing radiation dose. Nevertheless, the irradiated layer surfaces are not cracked or exfoliated; close and deep analysis of layer cross-sections showed their partial detachment from the substrate. However, the adherence tests performed by using the referenced adhesion tape did not indicate the delamination of the layers.

The EDS depth profiles and the atomic percentage curve analysis indicate the Si diffusion into the volumes of MgHApCs irradiated layers up to their surfaces. These data are confirmed by identifying Si 2p peak in the XPS spectra of irradiated layers. In addition, the XPS analysis ascertained that the electron beam irradiations do not alter the Mg-doped hydroxyapatite structure. XRD analysis has shown that MgHAp films irradiated at 50 Gy are better crystallized, which is in accordance with SEM and FTIR studies. 

The fitting FTIR spectral studies indicate the redistribution of phosphorous and oxygen atoms in the irradiated layers and the preservation of apatite and chitosan molecular structures. No additional IR absorption bands were observed besides Si-O bonds formation in the MgHApCs irradiated layers. Therefore, there are no molecular by-products from electron beam irradiations.

The results presented in this paper represent a first step in evaluating the effects of the exposure to electron beam (following radiotherapy treatment protocols for bone cancers) of Mg-doped hydroxyapatite/chitosan composite layers as a possible candidate for the covering of orthopedic prosthesis.

## Figures and Tables

**Table 1:** The assignment of IR absorption bands [13,14,36,37,38].

MgHApCs Sputtering Target		Unirradiated MgHApCs Layer		2 Gy Irradiated MgHApCs Layer		50 Gy Irradiated MgHApCs Layer	
Wave- Number [cm^−1^]	IR Band Assignment	Wave-Number [cm^−1^]	IR Band Assignment	Wave-Number [cm^−1^]	IR Band Assignment	Wave- Number [cm^−1^]	IR band Assignment
1118	P-O vibration in non-apatite phosphate structure	1155, 1128	P-O vibration in non-apatite phosphate structure	1160, 1131	P-O vibration in non-apatite phosphate structure	1131	P-O vibration in non-apatite phosphate structure
1090, 1075 1023, 936	P-O asymmetric and symmetric stretching vibrations in [PO_4_]^3−^ groups of the apatite structure	1086, 1055, 1026, 936	P-O asymmetric and symmetric stretching vibrations in [PO_4_]^3−^groups of the apatite structure	1085, 1055, 1037	P-O asymmetric and symmetric stretching vibrations in [PO_4_]^3−^groups of the apatite structure	1087, 1057, 1018, 938	P-O asymmetric and symmetric stretching vibrations in [PO_4_]^3−^groups of the apatite structure
991	C-H/C-O vibrations in chitosan	1008, 991	C-H/C-O vibrations in chitosan	997	C-H/C-O vibrations in chitosan	987	C-H/C-O vibrations in chitosan
				920	Si-OH vibrations	918	Si-OH vibrations
876	C-O vibrations in CO_3_^2−^/ P-O vibrations in HPO_4_^−^	886	C-O vibrations in CO_3_^2−^/ P-O vibrations in HPO_4_	889	C-O vibrations in CO_3_^2−^/ P-O vibrations in HPO_4_^−^	886	C-O vibrations in CO_3_^2−^/ P-O vibrations in HPO_4_^−^

## Data Availability

The data presented in this study are available on request from the corresponding author.
